# Hygroscopic behaviour and colour stability of 3D-printed and thermoformed aligner materials: an in vitro study

**DOI:** 10.1186/s12903-026-09977-x

**Published:** 2026-10-02

**Authors:** Jule Kolb, Sebastian Wille, Oral Cenk Aktas, Sinan Şen, Kathrin Puchert

**Affiliations:** 1 Department of Orthodontics, Christian-Albrechts University of Kiel, University Hospital Schleswig-Holstein, Campus Kiel, Kiel, Germany; 2 Department of Prosthodontics, Christian-Albrechts University of Kiel, University Hospital Schleswig-Holstein, Campus Kiel, Kiel, Germany

**Keywords:** Aligners, Orthodontics, 3D-printed aligners, Colour stability, Water sorption

## Abstract

**Background:**

Directly 3D-printed aligner materials have recently emerged as an alternative to established thermoformed clear aligners. The aim of this in vitro study was to investigate the colour stability, water sorption, and water solubility of directly 3D-printed aligner materials in comparison with an established thermoformed reference, as these properties are essential for clinical performance but currently insufficiently investigated for directly 3D-printed aligners.

**Methods:**

Tera Harz TC-85DAC, Freeprint Aligner, Freeprint Aligner with digital polishing, Dental LT Clear V2 and one thermoformed polyethylene terephthalate glycol material (Biolon) were examined. Water sorption and solubility were determined after seven days in distilled water at 37 °C. Colour stability was assessed after 12 h of immersion at 37 °C in turmeric, coffee, black tea, orange juice and distilled water. Total colour differences (ΔE*) were calculated spectrophotometrically using the Commission Internationale de l’Éclairage L*a*b* system and converted into National Bureau of Standards units. Data were analysed by one-way ANOVA with Games-Howell post hoc test and by Kruskal-Wallis test with Dunn’s test and Bonferroni correction (*p* < 0.05).

**Results:**

Biolon exhibited the lowest water sorption (mean 10.70 µg/mm³) and negligible solubility (median 0.00 µg/mm³). Among the printed resins, Tera Harz TC-85DAC showed the lowest water sorption (mean 17.28 µg/mm³) and solubility (median 2.50 µg/mm³), whereas Dental LT Clear V2 displayed the highest sorption (mean 29.53 µg/mm³) and Freeprint Aligner with digital polishing the highest solubility (median 17.38 µg/mm³). Turmeric caused the most pronounced discolouration, followed by coffee and black tea, while orange juice and water produced minimal changes. The thermoformed material remained most colour-stable (mean of the median ΔE* values across all immersion media = 4.21), whereas Dental LT Clear V2 showed the greatest discolouration (mean of the median ΔE* values = 27.35). Among the printed resins, Tera Harz TC-85DAC displayed the lowest staining susceptibility.

**Conclusions:**

The tested aligner materials differed markedly in water sorption, solubility, and colour stability. The thermoformed polyethylene terephthalate glycol material consistently showed the most favourable profile. Among the printed resins, Tera Harz TC-85DAC exhibited the most favourable combination of the investigated properties, supporting its potential for clinical application.

## Background

Clear aligner therapy has rapidly become a popular treatment option in orthodontics, largely due to the increasing demand among adult patients for aesthetically discreet alternatives to traditional fixed braces [1]. Compared with traditional fixed appliances, clear aligners offer superior aesthetics, greater patient comfort, and facilitated oral hygiene [2].

Conventionally, clear aligners are fabricated by thermoforming thermoplastic materials, most commonly polyethylene terephthalate glycol (PETG) and thermoplastic polyurethane (TPU), over 3D-printed staging models [3, 4]. However, this process entails non-uniform material thickness as well as considerable time, cost, and material requirements. Direct 3D printing eliminates the need for thermoforming and intermediate models, thereby reducing production steps, costs, and material waste [5].

Colour stability represents a particularly important property for clear aligners, as discolouration compromises their primary aesthetic advantage. Ideally, the optical properties should be preserved throughout the typical wear period of one to two weeks, during which aligners are worn 20–22 h per day [6]. Although patients are instructed to remove their aligners before consuming anything other than water, compliance has been reported to be insufficient [7], exposing the material to chromogenic substances. The extent of discolouration additionally depends on the chemical composition of the aligner material and the fabrication method [8]. As directly 3D-printed aligners introduce new resin-based compositions into clinical use, evaluating their colour stability relative to conventional thermoformed materials is essential.

Beyond colour stability, water sorption and solubility are critical determinants of clinical performance. Absorbed water acts as a plasticiser, promoting swelling and gradual degradation of the polymer network, which may impair dimensional stability, fit, and force transmission [9]. Water solubility reflects the release of unreacted monomers and leachable components [10], raising concerns regarding biocompatibility and material longevity. Importantly, water sorption and colour stability are closely interrelated, as increased water uptake enhances the diffusion of chromogenic agents into the bulk material [11].

A substantial body of literature has addressed these properties for thermoformed aligner materials, with PETG-based aligners consistently demonstrating high resistance to discolouration [11, 12] and favourable hygroscopic behaviour [13, 14]. In contrast, comparable data for directly 3D-printed aligner resins are markedly scarce. Although such materials are already in clinical use, systematic investigations of their optical and hygroscopic behaviour remain limited. Available studies indicate that printed aligner resins tend to exhibit clinically relevant discolouration after exposure to pigmenting media [15, 16] and that their water sorption may exceed that of thermoformed reference materials in the early immersion phase [17]. Studies investigating colour stability together with water sorption and solubility in directly 3D-printed orthodontic polymers are very rare. Neoh et al. addressed both aspects, although their investigation focused on retainer rather than aligner materials [9].

Despite these preliminary findings, fundamental gaps remain. For the majority of currently available aligner resins, data on optical and hygroscopic behaviour are either absent or limited to isolated materials.

Against this background, the present in vitro study aimed to investigate the water sorption, water solubility, and colour stability of four 3D-printed aligner materials, one of which received Medical Device Regulation (MDR; Regulation (EU) 2017/745) certification only at the end of January 2026, in comparison with an established thermoformed reference material.

Based on the experimental design, three null hypotheses were formulated: The first null hypothesis (H1₀) stated that there are no significant differences in water sorption and water solubility between the tested aligner materials. The second null hypothesis (H2₀) stated that the staining solutions cause comparable colour changes within a given material, and the third null hypothesis (H3₀) stated that all materials exhibit comparable colour changes within a given staining solution.

## Methods

### Materials

In this study, four 3D-printed resin materials (Tera Harz TC-85DAC (TH), Freeprint Aligner (FA), Freeprint Aligner with digital polishing (FA dp), and Dental LT Clear V2 (LT)) and one thermoformed material (Biolon (B)) were investigated (Table 1). Although LT is intended by the manufacturer for occlusal splints rather than clear aligners, it was included in this study because previous work has indicated its potential use in the direct fabrication of orthodontic aligners [18–20]. At the time of testing, the FA resin constituted a developmental material and was certified in late January 2026 under the designation dx direct aligner (Detax, Ettlingen, Germany) as a Class IIa medical device in accordance with the Medical Device Regulation (MDR; Regulation (EU) 2017/745). According to the manufacturer, the marketed dx direct aligner is chemically identical to the tested developmental resin.

**Table 1:** Overview of tested thermoformed and 3D-printed materials with fabrication and composition data

Material fabrication	Material	Device	Printing technology	Processing parameters	Material composition	Batch number
3D printing	Tera Harz TC-85DAC (TH) (Graphy, Seoul, South Korea)	NBEE (UNIZ Technology LLC, San Diego, CA, USA)	LCD-based stereolithography (LCD-SLA)	Excess resin removed manually; post-cured in Tera Harz Cure 2 (Graphy, Seoul, South Korea) for 20 min under nitrogen atmosphere	Light-curable aliphatic polyester-urethane with vinyl/(meth)acrylate end groups	1-D0110C11
	Freeprint Aligner (FA) (Detax GmbH, Ettlingen, Germany)	Max (Asiga, Alexandria, NSW, Australia)	digital light processing (DLP)	Two ultrasonic cycles in isopropanol; post-cured in Otoflash G171 with 2 × 2000 flashes under nitrogen atmosphere, followed by 24 h at 60 °C in an oven	Light-curable polyurethane-based (meth)acrylate	270398
	Freeprint Aligner digital polished (FA dp) (Detax GmbH, Ettlingen, Germany)	Max (Asiga, Alexandria, NSW, Australia)	DLP	Two ultrasonic cycles in isopropanol; post-cured in Otoflash G171 (NK-Optik GmbH, Baierbrunn, Germany) with 2 × 2000 flashes under nitrogen atmosphere, followed by 24 h at 60 °C in an oven	Light-curable polyurethane-based (meth)acrylate	270398
	Dental LT Clear V2 (LT) (Formlabs Inc., Somerville, MA, USA)	Form 3B+ (Formlabs Inc., Somerville, MA, USA)	Low force stereolithography (LFS)	Form Wash (Formlabs Inc., Somerville, MA, USA): 15 min in isopropanol followed by 5 min in fresh isopropanol; air-dried for 30 min at room temperature; post-cured in Form Cure (Formlabs Inc., Somerville, MA, USA) at 60 °C for 60 min	Light-curable aromatic-aliphatic dimethacrylate-based polymer (mainly Bisphenol A dimethacrylate and urethane dimethacrylate, with methacrylate monomers and photoinitiators)	DC01240129-01
Thermoforming	Biolon (B) (Dreve Dentamid GmbH, Unna, Germany)	Drufomat Scan (Dreve Dentamid GmbH, Unna, Germany)	-	Heating time: 1:10 min (0.625 mm foil) / 1:15 min (0.75 mm foil); cooling time: 1 min	Polyethylene terephthalate glycol (PETG)	3787842X 3 3790705X1

### Specimen preparation

**Fig. 1:**
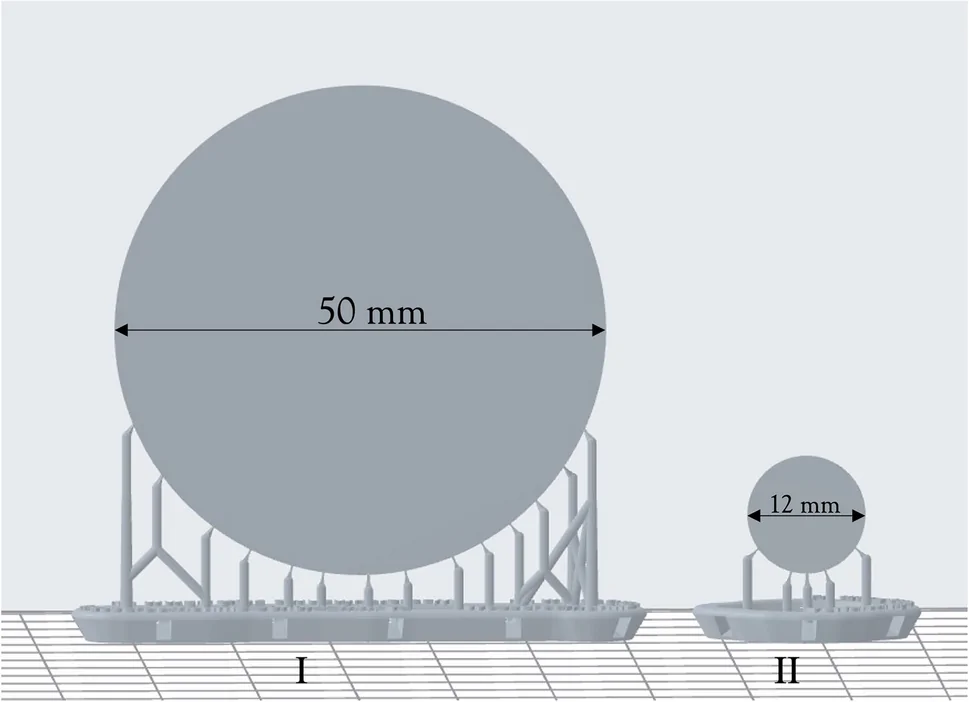
Printing setup as displayed in PreForm (Formlabs): water sorption and water solubility test (I), colour stability test (II)

All specimens were printed with a layer thickness of 100 μm. The specific printing orientation for each specimen type used in the various experiments is illustrated in Fig. 1. The material-specific printing, thermoforming, and post-processing protocols are summarised in Table 1. All specimens were fabricated according to the respective manufacturer’s protocol: TH specimens by Forestadent (Pforzheim, Germany), FA and FA dp specimens by Detax (Ettlingen, Germany), and LT and B specimens in-house. For FA and FA dp, the identical resin was used; the two variants differed exclusively in the printing tray: a universal tray for FA and an UltraGLOSS tray (Asiga, Alexandria, NSW, Australia) for FA dp.

For the determination of water sorption and solubility, specimens with a diameter of 50 mm and a thickness of 0.5 mm were fabricated in accordance with ISO 20795-2:2013 [21]. For colour stability testing, specimens with a diameter of 12 mm and a thickness of 0.6 mm were produced, as a thickness of 0.6 mm represents a commonly used material thickness for clear aligners. The 3D-printed specimens were fabricated directly in these dimensions, whereas thermoforming foils of 0.625 mm and 0.75 mm thickness were used for the thermoformed specimens. Due to material thinning during the thermoforming process, the final thicknesses of the thermoformed specimens corresponded to those of the 3D-printed specimens (0.5 mm and 0.6 mm, respectively). The final thickness of each specimen was measured individually using a digital micrometer (Filetta, Schut Geometrische Messtechnik GmbH, Trossingen, Germany). After removal of the support structures, all 3D-printed specimens were smoothed at the attachment areas using Liskoid polishing discs (Erkodent Erich Kopp GmbH, Pfalzgrafenweiler, Germany). While the manufacturers Graphy and Detax do not recommend additional surface polishing for their aligner materials, Formlabs specifies a polishing procedure in its manufacturing guide. Therefore, specimens made from LT were additionally polished using pumice powder (Ernst Hinrichs Dental GmbH, Goslar, Germany) followed by universal polishing paste (Ivoclar Vivadent AG, Schaan, Liechtenstein).

**Fig. 2:**
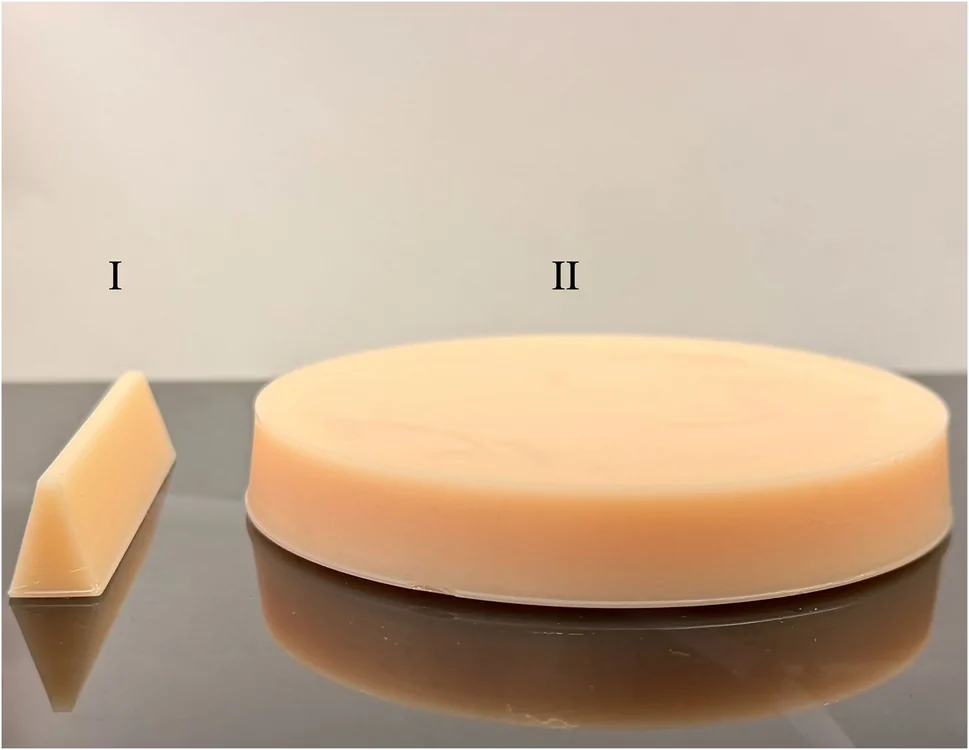
3D printed thermoforming molds for specimen preparation: trapezoidal prism (I) and truncated cone (II). The trapezoidal prism was used to prepare specimens for colour stability testing. The truncated cone served for specimen fabrication for water sorption and solubility testing

For the thermoforming process, two 3D molds were designed: a trapezoidal prism with the general geometry inspired by the design of Ryu et al. [13], and a truncated cone (Fig. 2). These molds were printed from Model Resin V3 (Formlabs Inc., Somerville, MA, USA). The horizontal surface of the 0.625 mm foils thermoformed over the truncated cone mold was used for preparing the specimens for water sorption and solubility testing, whereas the inclined side surfaces of the 0.75 mm foils thermoformed over the trapezoidal prism were used for colour stability testing. The specimens were prepared by cutting them from the thermoformed foils.

Sample size calculations were performed using G*Power (version 3.1.9.7) with a significance level of α = 0.05 and a statistical power of 80%. For the colour stability test, an effect size of f = 0.4 was applied based on the findings of Perumalla et al. [16], yielding a required total sample size of *N* = 175. Given the experimental design involving 25 groups, defined by the combination of five materials and five staining solutions, this corresponds to a minimum of seven specimens per group. To increase statistical robustness, ten specimens were included per group, resulting in a total of 250 specimens. For the water sorption and water solubility tests, a larger effect size of f = 0.6 was assumed based on the low standard deviations and distinct group differences reported by Albilali et al. [22] as well as the higher water sorption and solubility values of 3D-printed compared to thermoformed retainer materials observed by Neoh et al. [9]. The analysis indicated a minimum of eight specimens per group. To enhance statistical reliability and account for potential measurement variability, ten specimens per group were used, resulting in *N* = 50.

### Testing

#### Water sorption and solubility

All procedures were conducted in accordance with ISO 20795-2:2013 [21].

**Fig. 3:**
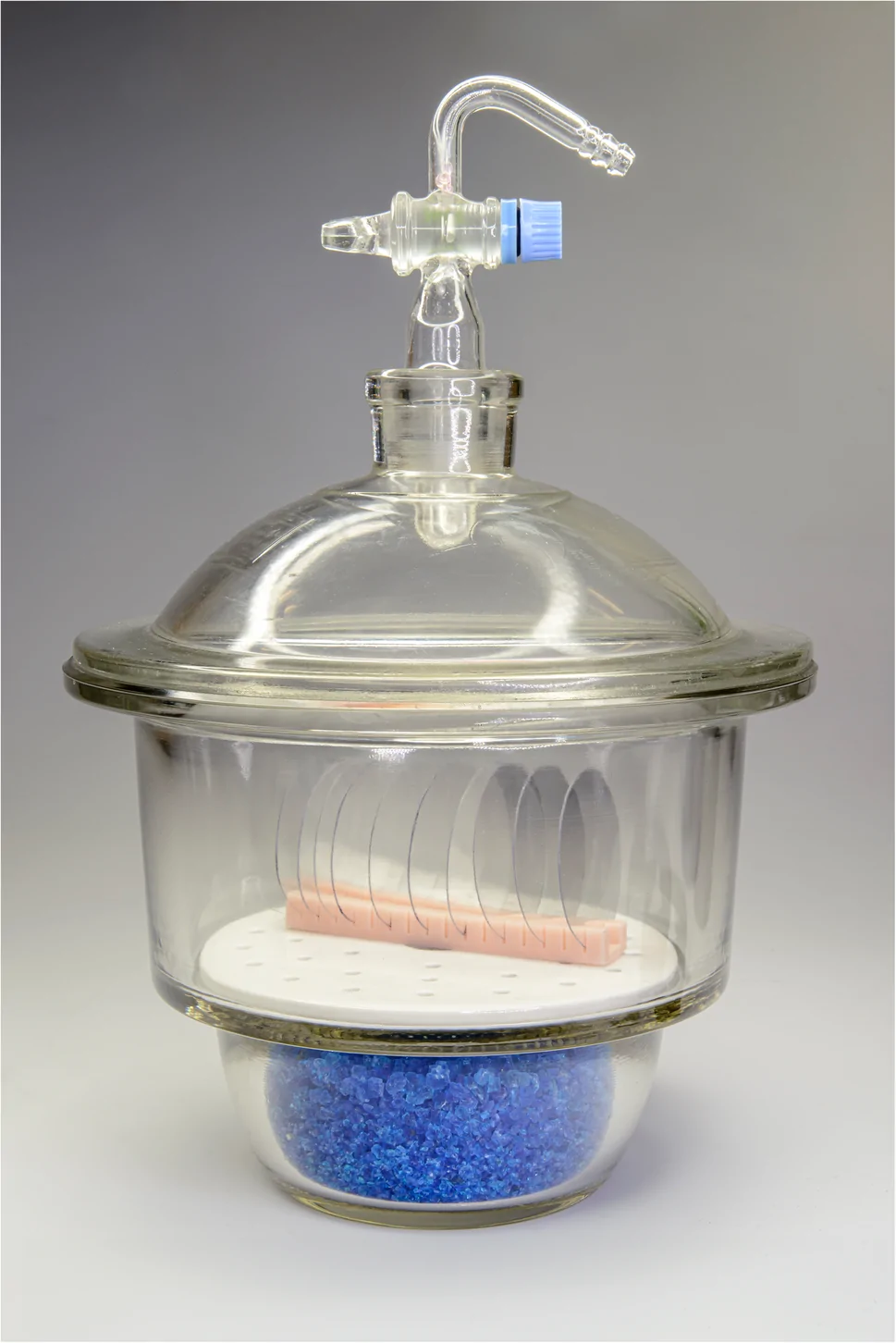
Desiccator used for specimen drying prior to weighing

The specimens were first dried in a desiccator for 23 ± 1 h at 37 ± 1 °C in a laboratory incubator (Incu-Line, VWR International GmbH, Darmstadt, Germany) and subsequently transferred to a second desiccator maintained at 23 ± 2 °C (Fig. 3). After 60 ± 10 min, the specimens were weighed using an analytical balance (A 200 S, Sartorius AG, Göttingen, Germany). The drying and weighing cycle was repeated until a constant mass (m₁) was obtained. The specimen volume (V) was calculated accordingly.

The conditioned specimens were then immersed in distilled water at 37 ± 1 °C for 7 days ± 2 h (Thermo Scientific Precision GP 02 water bath, Fisher Scientific GmbH, Schwerte, Germany). After removal, the specimens were blotted dry with a towel, gently waved in air for 15 ± 1 s, and weighed again 60 ± 10 s after removal (m₂). Subsequently, the specimens were re-dried under the same conditions as initially described until a constant reconditioned mass (m₃) was achieved.

Water sorption (wsp) and water solubility (wsl) were calculated using the following equations:

$$\mathrm{w}_\mathrm{sp} [\mu \mathrm{g}/\mathrm{mm}^3] = \frac{\mathrm{m}_2 - \mathrm{m}_1} {\mathrm{V}}$$

$$\mathrm{w}_\mathrm{sl} [\mu \mathrm{g}/\mathrm{mm}^3] = \frac{\mathrm{m}_1 - \mathrm{m}_3} {\mathrm{V}}$$

#### Colour stability

Four staining solutions were prepared according to the compositions shown in Table 2, with distilled water serving as the control medium.

**Table 2:** Composition of staining solutions

Staining solution	Brand	Preparation protocol
Distilled water (control)	-	1.5 L distilled water undiluted (as is)
Orange juice	Klindworth Osa Orange (Niehoffs Vaihinger Fruchtsaft GmbH, Lauterecken, Germany)	1.5 L orange juice undiluted (as is)
Black tea	Ostfriesen Teefix schwarzer Tee (Teekanne GmbH & Co. KG, Düsseldorf, Germany)	four tea bags steeped in 1.5 L boiling distilled water for 2–5 min
Coffee	Nescafé Classic (Nestlé S.A., Vevey, Switzerland)	10 teaspoons of instant coffee diluted in 1.5 L of boiling distilled water
Turmeric	Achterhof (Blank’s GmbH & Co. KG, Bunde, Germany)	1.5 g turmeric powder diluted in 1.5 L distilled water

The specimens were immersed in the respective staining solutions for 12 h at 37 °C. This period approximates the cumulative effect of two weeks of regular coffee consumption [23] and thus the typical wear period of an aligner before replacement.

Immersion was performed in a Julabo Mini water bath (JULABO GmbH, Seelbach, Germany) equipped with a custom-built stirring system to ensure uniform mixing of the solutions and to prevent the formation of precipitates.

During immersion, the specimens were placed in a custom-designed holder that allowed for parallel alignment with the stirring motion, ensuring that each specimen remained separated and was uniformly exposed to the solution.

Colour measurements were performed using the CIE L*a*b* colour system established by the Commission Internationale de l’Éclairage (CIE). In this system, the colour parameter L* represents lightness ( + = lighter, * -* = darker), a* represents the red-green axis ( + = red, * -* = green), and b* represents the yellow-blue axis ( + = yellow, * -* = blue) [24].

Measurements were conducted before immersion (T₀) and after 12 h of storage (T₁) using a spectrophotometer (VITA Easyshade, VITA Zahnfabrik, Bad Säckingen, Germany). For each specimen, three repeated measurements were performed, with the optical probe tip positioned perpendicularly to the flat specimen surface.

A milled ceramic cylinder made of KATANA™ Zirconia STML in shade A2 was used as a background reference. The selection of this shade was based on previous studies [15, 25], in which it was chosen due to its high prevalence among young adults.

Before each measurement, the specimens were rinsed with distilled water and dried with oil-free compressed air. All measurements were carried out under standardised lighting conditions in a darkened room.

The total colour difference (ΔE*) was calculated according to the following equation [24]:

$$\triangle \mathrm{E}^* = [ \left( \triangle \mathrm{L}^* \right)^2 + \left( \triangle \mathrm{a}^* \right)^2 + \left( \triangle \mathrm{b}^* \right)^2]^{1/2}$$

where ΔL*, Δa*, and Δb* represent the differences in the corresponding colour parameters between the two measurement times (T₁-T₀).

To provide a qualitative categorisation of perceptibility, the ΔE* values were converted into National Bureau of Standards (NBS) units using the following relationship:

$$\mathrm{NBS} = 0.92 \times \triangle \mathrm{E}^*$$

This conversion allows for classification of colour changes into visually perceptible levels:

- Trace (0.0-0.5),
- Slight (0.5–1.5),
- Noticeable (1.5-3.0),
- Appreciable (3.0–6.0),
- Much (6.0–12.0), and.
- Very much (≥ 12.0; change to another colour) [24].

### Statistical analysis

Statistical analyses were performed using SPSS software, version 29 (IBM Corp., Armonk, NY, USA). The Shapiro-Wilk test was applied to all experimental groups to assess the normality of data distribution.

For the experiments on water sorption and solubility, all datasets met the assumption of normal distribution (*p* > 0.05), except for the thermoformed samples in the water solubility test (*p* = 0.001). Homogeneity of variances was evaluated using Levene’s test (*p* < 0.001). A one-way analysis of variance (ANOVA) was performed to identify differences among the materials. Pairwise comparisons were conducted using the Games-Howell post hoc test, which provides robust results even when variances are unequal.

In the colour stability experiment, the Shapiro-Wilk test indicated significant deviations from normality in most groups. Therefore, nonparametric methods were applied. The Kruskal-Wallis test was used to evaluate differences in ΔE* values both within a single material across different staining solutions and within a staining solution across different materials. When significant differences were found, pairwise comparisons were carried out using Dunn’s test with Bonferroni correction.

Results are presented as mean values with standard deviation and median values, with the primary descriptive measure indicated by the placement of the superscript letters in the respective tables. For all statistical analyses, the level of significance was set at *p* < 0.05.

## Results

### Water sorption and solubility

The results for water sorption and water solubility are presented in Tables 3 and 4. Significant overall differences among the tested materials were observed for both water sorption (*p* < 0.001, η^2^ = 0.993) and water solubility (*p* < 0.001, η^2^ = 0.962). Pairwise comparisons for water sorption revealed significant differences between all materials (*p* < 0.001), except for FA and FA dp (*p* = 0.480). For water solubility, significant differences were found between all material pairs (*p* < 0.001). The thermoformed material exhibited the lowest water sorption and negligible water solubility. Among the 3D-printed materials, TH demonstrated the lowest values for both parameters. LT showed the highest water sorption, whereas FA dp exhibited the highest water solubility.

**Table 3:** Water sorption of the tested materials after one week of water immersion

Material		Water sorption (µg/mm^3^)
B	Mean ± SD	10.70 ± 0.25^a^
	95% CI of the mean	10.52–10.88
	Median	10.72
TH	Mean ± SD	17.28 ± 0.32^b^
	95% CI of the mean	17.05–17.50
	Median	17.31
FA	Mean ± SD	23.30 ± 0.35^c^
	95% CI of the mean	23.05–23.56
	Median	23.25
FA dp	Mean ± SD	23.91 ± 1.08^c^
	95% CI of the mean	23.14–24.68
	Median	24.09
LT	Mean ± SD	29.53 ± 0.44^d^
	95% CI of the mean	29.21–29.84
	Median	29.36

Different superscript letters indicate statistically significant differences (*p* < 0.05, Games-Howell test)

*SD* standard deviation, *CI* confidence interval

**Table 4:** Water solubility of the tested materials after one week of water immersion

Material		Water solubility (µg/mm^3^)
B	Median	0.00^a^
	Mean ± SD	0.07 ± 0.09
	95% CI of the mean	0.00–0.13
TH	Median	2.50^b^
	Mean ± SD	2.46 ± 0.21
	95% CI of the mean	2.32–2.61
FA	Median	12.99^d^
	Mean ± SD	12.55 ± 2.54
	95% CI of the mean	10.73–14.36
FA dp	Median	17.38^e^
	Mean ± SD	17.22 ± 1.55
	95% CI of the mean	16.10–18.33
LT	Median	5.27^c^
	Mean ± SD	5.24 ± 0.40
	95% CI	4.95–5.53

Different superscript letters indicate statistically significant differences (*p* < 0.05, Games-Howell test)

*SD* standard deviation, *CI* confidence interval

### Colour stability

**Fig. 4:**
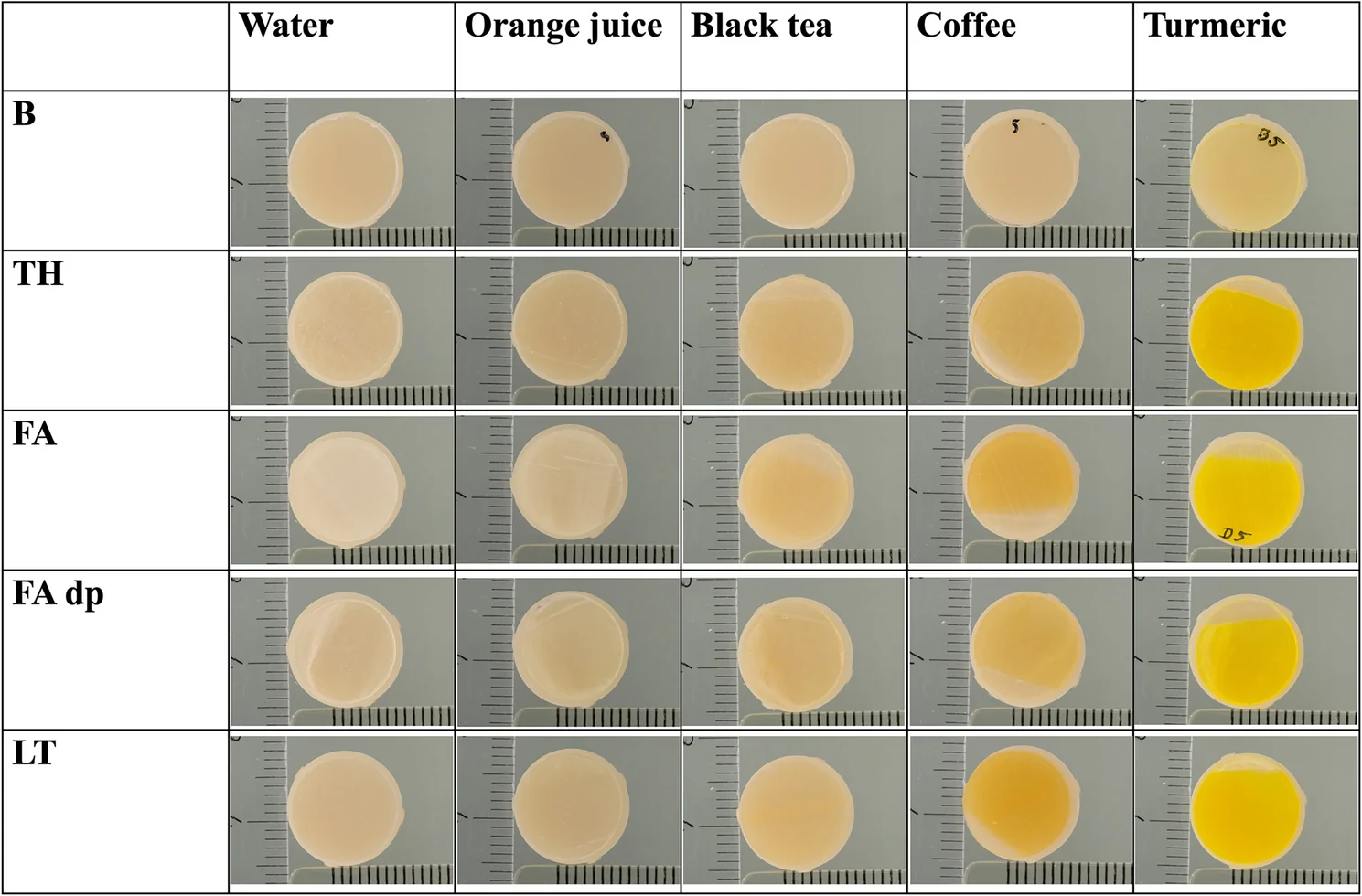
Representative photographs of all material-immersion medium combinations after 12 h of immersion. Rows represent the tested materials, while columns represent the respective immersion media, with water serving as the reference medium

The colour stability analysis revealed significant differences in both the susceptibility of the materials to discolouration and the staining potential of the solutions (Kruskal-Wallis test, *p* < 0.001). Effect sizes were η^2^_H_ = 0.247–0.922 for differences between materials within each staining solution and η^2^_H_ = 0.907–0.959 for differences between staining solutions within each material (Table 5). The corresponding NBS categories for each material-staining solution combination are presented in Table 6. A visual overview of the colour changes observed in all material-solution combinations is provided in Fig. 4. Across all materials, turmeric induced the most pronounced colour deviations (median ΔE* 16.65–79.82; NBS category “very much”), followed by coffee, black tea, orange juice, and water. Coffee led to distinct discolourations (“very much”) in all 3D-printed materials, while black tea only partially reached this category. Orange juice produced smaller changes than coffee, tea, and turmeric, although some printed materials exceeded the clinical acceptability threshold.

Significant material-dependent differences were also observed within each staining solution (*p* < 0.001). The thermoformed material B exhibited the highest overall colour stability (mean of the median ΔE* values across all immersion media = 4.21), remaining within the “trace” category in water and orange juice and within the “noticeable” category in coffee and black tea. Only turmeric produced distinct discolourations (“very much”).

Among the 3D-printed materials, TH exhibited the lowest discolouration tendency (mean of the median ΔE* values across all immersion media = 19.58), performing significantly better than LT and both FA variants. LT showed the greatest overall discolouration, with a mean of the median ΔE* values across all immersion media of 27.35. The two FA materials did not differ significantly from each other.

**Table 5:** ΔE*-values of the tested materials exposed to different staining solutions

Material		Water	Orange juice	Black tea	Coffee	Turmeric	Effect size η^2^_H_
B	Median	0.25^aA^	0.32^aA^	1.16^aBC^	2.69^aCD^	16.65^aD^	0,907
	Mean ± SD	0.35 ± 0.23	0.33 ± 0.13	1.16 ± 0.26	2.66 ± 0.32	18.58 ± 5.90	
	95% CI of the mean	0.26–0.43	0.28–0.38	1.06–1.26	2.55–2.78	16.38–20.78	
TH	Median	0.72^bA^	1.46^aA^	8.03^aBC^	13.98^aCD^	73.71^bD^	0,932
	Mean ± SD	0.83 ± 0.53	1.45 ± 0.16	8.69 ± 1.26	13.80 ± 0.79	73.62 ± 1.29	
	95% CI of the mean	0.63–1.03	1.39–1.51	8.22–9.16	13.50–14.09	73.13–74.10	
FA	Median	1.09^bA^	3.22^bA^	17.25^bBC^	28.15^bcC^	79.11^cD^	0,915
	Mean ± SD	1.59 ± 1.39	3.35 ± 0.49	19.60 ± 5.57	27.98 ± 2.82	79.14 ± 1.68	
	95% CI of the mean	1.07–2.11	3.17–3.54	17.52–21.68	26.93–29.04	78.51–79.77	
FA dp	Median	0.80^bA^	3.41^bA^	17.38^bBC^	22.85^bC^	79.82^cD^	0,938
	Mean ± SD	0.88 ± 0.46	3.51 ± 1.06	17.17 ± 3.80	22.15 ± 2.36	78.87 ± 1.85	
	95% CI of the mean	0.71–1.05	3.11–3.91	15.75–18.59	21.27–23.03	78.18–79.56	
LT	Median	0.55^bA^	3.67^bAB^	20.99^bBC^	33.82^cCD^	77.72^cD^	0,959
	Mean ± SD	0.66 ± 0.36	3.72 ± 0.41	20.33 ± 2.65	33.32 ± 3.06	77.47 ± 1.52	
	95% CI of the mean	0.53–0.79	3.57–3.87	19.34–21.32	32.17–34.46	76.90–78.04	
Effect size η^2^_H_		0,247	0,787	0,779	0,922	0,786	

Pairwise comparisons of staining solutions within each material: different superscript uppercase letters indicate significant differences (Dunn test with Bonferroni correction, *p* < 0.05). Pairwise comparisons of materials within each staining solution: different superscript lowercase letters indicate significant differences (Dunn test with Bonferroni correction, *p* < 0.05)

*SD* standard deviation, *CI* confidence interval

**Table 6:** NBS categories with ΔE* intervals and corresponding material-staining solution combinations

NBS category	ΔE* intervals	NBS units	Material-staining solution combinations
Trace	≤ 0.54	0.0-0.5	B-Water B-Orange juice
Slight	0.55–1.63	0.5–1.5	B-Black tea TH-Water TH-Orange juice FA-Water FA dp-Water LT-Water
Noticeable	1.64–3.26	1.5-3.0	B-Coffee FA-Orange juice
Appreciable	3.27–6.52	3.0–6.0	FA dp-Orange juice LT-Orange juice
Much	6.53–13.04	6.0–12.0	TH-Black tea
Very much; change to another colour	≥ 13.05	≥ 12.0	B-Turmeric TH-Coffee TH-Turmeric FA-Black tea FA-Coffee FA-Turmeric FA dp-Black tea FA dp-Coffee FA dp-Turmeric LT-Black tea LT-Coffee LT-Turmeric

## Discussion

The present study investigated the water sorption, solubility, and colour stability of four 3D-printed aligner materials: TH, LT, as well as FA and FA dp, the latter two representing two printing variants of the same resin. An established thermoformed material (B) served as the reference. The study aimed to address the current lack of systematic comparative data on the optical and hygroscopic behaviour of directly 3D-printed aligner resins, which are already in clinical use but remain insufficiently characterised in the literature. As the FA resin constituted a developmental material at the time of testing and was only certified as a medical device at the end of January 2026, no externally acquired data on the aforementioned properties of this resin have, to the best of current knowledge, been published to date. To the authors’ knowledge, the present work represents the first independent in vitro characterisation of this now-approved material.

A methodological strength to be emphasised is the compliance with ISO 20795-2:2013 [21]. Although this standard was originally developed for orthodontic base polymers rather than specifically for aligner materials, no dedicated testing standard currently exists for this purpose. The standardised specifications regarding specimen geometry, conditioning, and measurement intervals ensure reproducibility and comparability with other investigations conducted in accordance with the standard.

The null hypothesis H1₀ was rejected, as the materials differed significantly with respect to water sorption and solubility. The printed resins exhibited significantly higher water sorption than the thermoformed reference material. Among the 3D-printed materials, TH showed the lowest values, followed by FA and FA dp (without a significant difference), and LT with the highest values. The minimal difference of approximately 0.6 µg/mm³ between FA and FA dp indicates a largely unchanged bulk hydrophilicity and a comparable free volume. According to ISO 20795-2:2013 [21], water sorption should not exceed 32 µg/mm³. All materials remained below this threshold and thus fulfilled the minimum requirements for dental polymers with respect to water sorption. As this threshold originates from orthodontic base polymers and was not established specifically for aligner materials, compliance should be regarded as a reference benchmark rather than as evidence of clinical suitability.

For water solubility, significant differences were observed between all materials. The thermoformed material exhibited virtually no measurable water solubility, whereas the 3D-printed materials showed median values between 2.5 and 17.38 µg/mm³. The ISO limit of 5 µg/mm³ [21] was slightly exceeded by LT (+ 0.27 µg/mm³) and markedly exceeded by both FA variants. Only TH remained below this limit. However, the limitations noted above regarding the applicability of this ISO threshold to aligner materials should also be considered. Notably, FA dp displayed median values approximately 34% higher than FA, indicating an increased release of soluble constituents from the digitally polished material.

The leached substances could potentially exert cytotoxic effects within the intraoral environment [26]. For TH, in vitro data on human gingival fibroblasts show only a transient, non-cytotoxic exposure [27]. However, under prolonged pre-incubation in artificial saliva and at higher extract concentrations, cytotoxic effects have been reported despite generally preserved biocompatibility [28]. The evidence therefore remains inconsistent.

A further notable observation is the discrepancy between the manufacturer’s specification for FA (< 5 µg/mm³; ISO 20795-2:2013) [29] and the median values determined in this study of 12.99 µg/mm³ (FA) and 17.38 µg/mm³ (FA dp). Since the specimens were fabricated by the manufacturer under a validated printing and post-curing protocol, and no formulation changes occurred between the developmental and the market version, process- and formulation-related causes appear unlikely as the primary explanation. Batch-related variation represents one possible explanation, although this cannot be confirmed based on the present experimental design. However, storage- and transport-related influences cannot be completely excluded, as the specimens were not tested immediately after manufacturing but underwent postal shipment before testing. In contrast, the measured values for B (safety data sheet: practically insoluble [30]) and TH (manufacturer specification 2.0 µg/mm³ [31]) were in close agreement, supporting the methodological validity of the testing procedure.

Interestingly, water sorption and solubility did not follow a consistent pattern across materials: while LT exhibited the highest sorption, the highest solubility was observed for FA dp. This dissociation suggests that both parameters are governed by distinct structural and processing factors rather than a single underlying mechanism.

The discrepancy in water solubility between FA and FA dp despite comparable water sorption may be explained by the printing process. Both variants differ exclusively in the vat type, while sharing identical resin formulation and post-processing. The digitally polished variant was printed using the UltraGLOSS vat, whose diffusing film optically masks layer-induced stair-step artefacts. The angular dispersion of incident light may alter exposure conditions at previously cured layers and affect interlayer bonding quality. As the layers, due to the 90° printing orientation, are oriented perpendicular to the flat specimen surfaces, more heterogeneous interlayer interfaces may facilitate increased release of low-molecular-weight constituents without substantially altering bulk hydrophilicity. However, this proposed explanation is specific to the manufacturing workflow and printing orientation investigated in the present study, and its applicability to other digital polishing systems or printing protocols requires further investigation.

Layer-by-layer photopolymerisation produces an inherent step structure, microporosities, and microchannels [17], which increase both the effective surface area and the diffusion pathways available to water. Residual monomers, oligomers, and photoinitiator fragments remaining from the manufacturing process may be leached out by penetrating water [10]. In contrast, thermoforming produces homogeneous, smooth surfaces [32] and is based on an already polymerised material with a stable polymer network and a low content of low-molecular-weight constituents.

The findings on water sorption are consistent with previous investigations. Šimunović et al. [17] examined two thermoformed multilayer aligner materials and two printed aligner materials with regard to water sorption during a 14-day immersion in distilled water at 37 °C. In the early immersion phase (days 1–7), the 3D-printed materials exhibited significantly higher water sorption than the thermoformed materials, which is consistent with the findings of the present study after 7 days. From day 8 onwards, this trend reversed, particularly for the material with an outer TPU layer, while the material with an outer PETG layer showed a more moderate increase. The pure PETG used in the present study most closely corresponds structurally to the outer layer of the multilayer material with an outer PETG layer, rendering the moderate water sorption values of B plausible.

Neoh et al. [9] likewise reported higher water sorption for 3D-printed retainers compared with PETG and TPU materials after 7 days. Among these thermoplastic materials, the PETG-based material exhibited both lower water sorption and lower water solubility than the TPU-based material, which is consistent with comparatively greater hydrophilicity of the TPU formulation. In that study, LT also showed the highest water sorption, although not the highest water solubility.

A similar pattern is oberserved for the thermoformed reference material. The mean values determined for B in the present study (water sorption: 10.70 µg/mm³; water solubility: 0.07 µg/mm³) fall within the range reported in the literature for PETG-based aligner sheets. Ryu et al. reported a water sorption of approximately 12 µg/mm³ and a solubility of about 2.8 µg/mm³ for a PETG-based aligner material after 14 days of water storage [13]. Inoue et al. observed water sorption values of 8.5 µg/mm³ after 24 h and 11.8 µg/mm³ after two weeks [14].

Colour stability was determined spectrophotometrically after 12 h of immersion at 37 °C in four staining solutions and distilled water as control. Comparable immersion times have also been employed in other studies on the colour stability of aligner materials [11, 12, 33]. Some studies employ significantly longer immersion periods of several days [15, 16], although such exposure durations only partially reflect clinical reality, as staining media such as orange juice, coffee, or tea act only briefly in the oral cavity. The duration chosen here approximates the cumulative effect of these media over a realistic wear period and thereby permits a clinically oriented interpretation of the results. While the NBS classification provides a qualitative categorisation of perceptibility, a ΔE* value ≥ 3.3 was additionally applied as the clinical acceptance threshold, above which discolourations are considered clinically unacceptable [34].

Statistical analysis revealed significant differences both between the staining solutions within each material and between the materials within each staining solution, leading to the rejection of H2₀ and H3₀.

Applying the clinical acceptance threshold of ΔE* ≥ 3.3 [34], the thermoformed reference material remained clinically acceptable in all staining solutions except turmeric, whereas all 3D-printed materials exceeded this threshold in black tea, coffee, and turmeric. Among the printed resins, TH displayed the most favourable profile, while LT was most prone to discolouration. The two FA variants did not differ significantly.

Although water sorption and solubility varied differently across the materials, some similarities were observed between the material-specific water sorption and staining susceptibility. B exhibited both the lowest water sorption and the highest colour stability, whereas LT, with the highest water sorption, also showed the most pronounced discolouration. TH ranked as the best-performing 3D-printed material in both parameters. These observations may indicate a potential relationship between water sorption and staining susceptibility, although this remains hypothetical. Water may act as a transport medium for dissolved chromophores and facilitate their diffusion into the polymer network [11]. Surface roughness has also been discussed as an influencing factor [9]. However, the present results suggest that it is not the dominant one. FA dp was printed using the UltraGLOSS tray, which produces an optically smoothed surface effect, whereas LT was mechanically polished according to manufacturer guidance. Both materials nevertheless showed the highest susceptibility to discolouration, while TH, without any additional surface treatment, exhibited the best colour stability among the 3D-printed materials. Moreover, no significant differences were observed between FA and FA dp. This suggests that material-dependent differences are primarily determined by the chemical composition and polymer structure, while surface roughness likely plays a subordinate role.

To date, only a few studies have investigated the colour stability of printed aligners or retainers with respect to curcumin or curry [9, 16], despite the widespread consumption of these substances, particularly in South Asian populations [35]. In agreement with the present study, higher ΔE* values were reported for these media than for coffee or black tea.

The present results are consistent with a growing body of evidence indicating a higher susceptibility to discolouration of printed as compared with thermoformed aligner materials. Neoh et al. [9] reported, for LT after 24-hour immersion in highly pigmented solutions (Thai curry, coffee, black tea), discolouration within the NBS category “very much”, whereas the PETG-based material exhibited clinically relevant changes only in Thai curry. This corresponds to the behaviour of LT and B observed in the present study. Among the thermoformed materials investigated by Neoh et al. [9], the PETG-based and the TPU-based materials showed similarly low discolouration in coffee, whereas the TPU-based material exhibited less pronounced colour changes than the PETG-based material following immersion in Thai curry. Similarly, Daniele et al. [36] reported that both PETG-based and TPU-based aligner materials exhibited colour changes within the NBS category “noticeable” after seven days of immersion in coffee. The findings for the PETG-based material are consistent with those of the present study, in which B likewise exhibited noticeable discolouration following coffee immersion.

Šimunović et al. [15] determined a ΔE* value of 17.24 (“very much”) for TH after 24 h in coffee, compared with 2.51 for the thermoformed material with an outer PETG layer (“noticeable”). In contrast, the thermoformed material with an outer PU layer exhibited a considerably higher ∆E* value of 10.64 (“much”) under the same conditions. In black tea, TH was consistently classified within the NBS category “much”. Wu et al. [37] also employed a 12-hour immersion period in coffee for TH and reported a ΔE* value of approximately 8.5 (NBS: “much”) at a layer thickness of 100 μm and a 90° printing orientation, which, in agreement with the present findings, falls within the clinically unacceptable range. Rueda et al. [38] reported a significantly improved colour stability following optical polishing after 28 days of static storage in coffee, whereas no significant differences between FA and FA dp emerged in the present study. This divergence is probably attributable to the considerably longer exposure duration and the static conditions employed by Rueda et al.

Despite the standardised experimental design, the study is subject to inherent limitations of in vitro investigations. Salivary enzymes, occlusal loading due to bruxism, and mechanical stress arising from insertion and removal, which may influence water sorption and colour stability in vivo, were not taken into account. The flat specimens furthermore do not reproduce the anatomically complex shape of actual aligners, limiting transferability. Although the chosen immersion duration approximates the cumulative exposure time to staining media, it does not account for patient-side cleaning measures. Clear aligners can be cleaned mechanically by brushing, chemically using specialised cleaning agents or cleaning tablets, or by combining both approaches. These cleaning methods may reduce staining to varying degrees depending on the cleaning protocol and aligner material [6, 39]. The results should therefore be interpreted as a worst-case scenario and may overestimate in vivo discolouration. Furthermore, the detailed chemical composition of most tested resins is not publicly available, limiting material-chemical interpretation. While total mass loss due to solubility was determined gravimetrically, the chemical identity of released substances remains unknown, requiring further analytical investigation. Finally, as LT was not explicitly designated as an aligner material at the time of investigation but was intended by the manufacturer for occlusal splints, the findings for this material should be interpreted with caution regarding their direct clinical applicability to clear aligner therapy. Additionally, colour differences were quantified using the CIELAB ∆E* formula rather than the CIEDE2000 (∆E_00_) formula. ISO/TR 28642:2016 [40] recommends either formula for colour measurement in dentistry, and ∆E* was used here owing to its historical acceptance in dental research and its comparability with the NBS classification and with previous studies employing the same metric [9, 15, 16, 37]. While ∆E_00_ has been reported to reflect human colour perception more accurately [41], ∆E* remains a widely used and practical measure for comparative colour stability assessments. Future studies could additionally report ∆E_00_ to allow a more perceptually oriented interpretation of the results.

The temporal coincidence between the completion of the investigations and the MDR certification of the tested FA resin as dx direct aligner (at the end of January 2026) gives the results immediate translational relevance, as it provides clinicians with independently generated comparative data for the first time to help them select materials.

From a functional perspective, the higher water sorption observed for all printed materials compared to the thermoformed reference material may be clinically relevant, as greater water uptake could increase susceptibility to plasticisation and swelling [9]. This could potentially affect the materials’ dimensional stability, fit and force transmission. However, the extend of water sorption varied among the printed materials, suggesting material-dependent differences in susceptibility to water-induced changes. These differences could become relevant during the typical one- to two-week aligner wear period. Similarly, the higher water solubility of the printed materials indicates greater release of soluble constitutes than from the thermoformed reference material, although the clinical relevance of these differences during the relatively short aligner wear period remains uncertain.

From an aesthetic perspective, the clinically unacceptable discolouration observed after only 12 h of immersion in coffee and black tea underlines the necessity of comprehensive patient education. Patients should be explicitly advised to remove aligners prior to the consumption of pigmenting beverages and foods. Discolouration reaching or exceeding the clinical acceptance threshold (∆E* ≥3.3) could negatively impact patient satisfaction, especially since aesthetics are one of the main advantages of clear aligners over traditional fixed braces and could be a key factor in patients’ choice of treatment. Therefore, noticeable staining during the typical one- to two-week wear period could reduce patients’ willingness to wear the aligners consistently, which could compromise treatment adherence and consequently affect treatment outcomes. With regard to material selection, the data suggest that for patients with high aesthetic demands or regular consumption of chromogenic beverages, thermoformed PETG-based materials remain an attractive option, whereas among the printed materials TH exhibited the most favourable profile.

The present findings should be verified in clinical in vivo studies. At the materials-chemistry level, further analytical investigations are required in order to identify the nature and quantity of eluted monomers, oligomers, and photoinitiator fragments and to conclusively assess their biological relevance. In addition, the extent to which the effect of optical polishing depends on the chosen printing orientation remains to be clarified. In the present study, a print orientation of 90° was selected in order to avoid support structures on the flat specimen surfaces. Future investigations could address the influence of different printing angles and thereby contribute to the optimisation of process parameters for directly printed aligners.

## Conclusion

Within the limitations of this study, the following conclusions can be drawn:

1. The aligner materials investigated exhibited pronounced differences in water sorption, water solubility, and colour stability, with the thermoformed PETG-based material consistently achieving the most favourable values.
2. Directly printed aligner resins do not constitute a homogeneous material class. The pronounced inter-material variation in the hygroscopic and optical properties examined precludes generalisation across these resins and necessitates a material-specific assessment of clinical suitability.
3. Among the tested 3D-printed materials, TH showed the most favourable profile for the investigated properties.

## Data Availability

All data generated or analysed during this study are included in this published article.

## Abbreviations

B: Biolon
CIE: Commission Internationale de l’Éclairage
FA: Freeprint Aligner
FA dp: Freeprint Aligner with digital polishing
H1_0_: First null hypothesis
H2_0_: Second null hypothesis
H3_0_: Third null hypothesis
LT: Dental LT Clear V2
MDR: Medical Device Regulation; Regulation (EU) 2017/745
NBS: National Bureau of Standards
PETG: Polyethylene terephthalate glycol
PU: Polyurethane
SD: Standard deviation
TH: Tera Harz TC-85DAC
TPU: Thermoplastic polyurethane
ΔE*: Total colour difference
